# Consumption of Soft Drinks and Overweight and Obesity Among Adolescents in 107 Countries and Regions

**DOI:** 10.1001/jamanetworkopen.2023.25158

**Published:** 2023-07-24

**Authors:** Huan Hu, Jing Song, Graham A. MacGregor, Feng J. He

**Affiliations:** 1 Research Center for Prevention from Radiation Hazards of Workers, National Institute of Occupational Safety and Health, Kanagawa, Japan; 2 Wolfson Institute of Population Health, Barts and The London School of Medicine and Dentistry, Queen Mary University of London, London, United Kingdom

## Abstract

**Importance:**

Soft drink consumption is associated with weight gain in children and adolescents, but little is known about the association between soft drink consumption and prevalence of the overweight and obesity in adolescents.

**Objective:**

To investigate the association of soft drink consumption with overweight and obesity in adolescents enrolled in school (hereafter, school-going adolescents) using country-level and individual-level data.

**Design, Setting, and Participants:**

This cross-sectional study used data from 3 cross-sectional studies including 107 countries and regions that participated in the Global School-Based Student Health Survey (2009-2017), the European Health Behavior in School-Aged Children study (2017-2018), and the US Youth Risk Behavior Survey (2019).

**Exposure:**

Daily soft drink consumption (consuming soft drinks 1 or more times per day or not).

**Main Outcome and Measure:**

Overweight and obesity defined by the World Health Organization Growth Reference Data.

**Results:**

Among the 107 countries and regions, 65 were low- and middle-income, and 42 were high-income countries and regions, with a total of 405 528 school-going adolescents (mean [SD] age, 14.2 [1.7] years; 196 147 [48.4%] males). The prevalence of overweight and obesity among adolescent students varied from 3.3% (95% CI, 2.6 to 4.1) in Cambodia to 64.0% (95% CI, 57.0 to 71.6) in Niue, and the prevalence of adolescent students consuming soft drinks 1 or more times per day varied from 3.3% (95% CI, 2.9 to 3.7) in Iceland to 79.6% (95% CI, 74.0 to 85.3) in Niue. There was a positive correlation between the prevalence of daily soft drink consumption and the prevalence of overweight and obesity (*R*, 0.44; *P* < .001). The pooled analysis using individual-level data also showed a statistically significant association between daily soft drink consumption and overweight and obesity (daily soft drink consumption vs nondaily soft drink consumption), with an odds ratio of 1.14 (95% CI, 1.08 to 1.21) among school-going adolescents.

**Conclusions and Relevance:**

In this study of 107 countries and regions, the prevalence of daily consumption of soft drinks was associated with the prevalence of overweight and obesity among adolescent students. Our results, in conjunction with other evidence, suggest that reducing soft drink consumption should be a priority in combating adolescent overweight and obesity.

## Key Points

**Question:**

What is the association between soft drink consumption and prevalence of overweight and obesity in adolescents?

**Findings:**

In this cross-sectional study using data of 405 528 school-going adolescents (children enrolled in school) from 107 countries and regions, the prevalence of daily soft drink consumption was associated with the prevalence of overweight and obesity.

**Meaning:**

These findings suggest that reducing soft drink consumption is important for lowering overweight and obesity in adolescents, and action should be used to reduce the consumption of soft drinks.

## Teaser

This cross-sectional study examines the association of soft drink consumption with overweight and obesity in adolescents using both country-level and individual-level data in 107 countries and regions.

## Introduction

The consumption of soft drinks, particularly sugar-sweetened beverages, is associated with weight gain.^1^ In the past decades, soft drink consumption has increased in both high-income and low- and middle-income countries.^2,3^ Meanwhile, the prevalence of overweight and obesity in children, adolescents, and adults has also increased.^4^ A comprehensive understanding of the association between soft drink consumption and overweight and obesity is important for curbing the increasing trend of obesity, especially for low- and middle-income countries because many soft drink companies are stepping up their marketing and promotion of soft drink sales in these countries.^5,6^

Systematic reviews of cohort studies and experimental studies have provided a considerable amount of evidence that soft drink consumption is associated with weight gain in children and adolescents.^7,8,9^ However, there are only limited data on the association between soft drink consumption and country-level prevalence of overweight and obesity. One study in adults using data from 75 countries showed that every 1% increase in soft drink consumption was associated with a 4.8% increase in overweight and obesity.^3^ However, no such studies have been conducted in adolescents who are being increasingly targeted by the soft drink industry.^10^ Information about the role of soft drink consumption in the prevalence of overweight and obesity among adolescents is essential to prompt policy makers to prioritize action to reduce soft drink consumption.

The main objective of this study is to investigate the association between the prevalence of adolescent students consuming soft drinks once daily or more and the prevalence of overweight and obesity across 107 countries and regions, using aggregate data obtained from national school-based surveys. Additionally, we analyzed individual-level data to investigate the association between daily soft drink consumption and overweight and obesity among adolescents enrolled in school (hereafter, school-going adolescents).

## Methods

This study used data from 3 school-based surveys: the Global School-Based Student Health Survey (GSHS), the European Health Behavior in School-Aged Children (HBSC [Europe]) study, and the US National Youth Risk Behavior Survey (YRBS [US]). These surveys were conducted with a representative sample of a national population and collected data on both soft drink consumption and overweight and obesity. The sampling methods and data collection were provided in eTable 1 in Supplement 1. This study followed the Strengthening the Reporting of Observational Studies in Epidemiology (STROBE) reporting guideline for cross-sectional studies. This study is exempt from ethics approval as the study used open-access data from previous studies in which informed consent has already been obtained by the investigators.

### GSHS

The GSHS is a collaborative project led by the World Health Organization (WHO) and the US Centers for Disease Control and Prevention (CDC). The GSHS is a school-based survey to measure and assess behavioral risk factors and protective factors primarily among students aged 13 to 17 years. Ethical approval was obtained from both a national government (the Ministry of Health or Education) and an institutional review board or ethics committee in each country. Verbal or written consent was also obtained from the participants and their parents or guardians. Details about the GSHS were published on the WHO website.^11^ In the present study, we used surveys conducted from 2009 to 2017, which included 2 questionnaire versions: 1 from 2009 to 2012 and another from 2013 to 2017. Both versions included identical questions about soft drink consumption, ensuring consistency in data collection throughout the study period. For countries with repeated surveys, the latest available data was used.

### HBSC (Europe)

The HBSC (Europe) study is a cross-national, school-based survey that investigates the health and well-being of adolescents across Europe and North America in collaboration with the WHO Regional Office for Europe. Ethical approval was obtained for this study from the University Ethics Boards or other relevant authorities associated with the research team in each country. Informed consent was obtained from the participants and their parents or guardians. The study has been conducted regularly since 1983 and 1984, using a standardized methodology as detailed in the HBSC (Europe) international study protocol.^12^ The present study used data from the most recent available survey conducted in 2017 and 2018.

### YRBS (US)

The YRBS (US) is a national school-based survey conducted every 2 years by the CDC to monitor health risk behaviors among US adolescents. The survey uses both active and passive written parental consent, following local parental permission procedures, and is approved by the CDC’s institutional review board. Students participate in the survey anonymously and voluntarily. Detailed information about the YRBS (US) has been reported elsewhere.^13^ Our study used the 2019 survey data, which was the latest data available during the research period.

As shown in eFigure 1 in Supplement 1, the study involved 107 countries and regions from the GSHS (n = 61), the HBSC (Europe) study (n = 45), and the YRBS (US) (n = 1). Adolescent students with incomplete data about soft drink consumption, overweight and obesity, and covariates were excluded, leaving a sample size of 405 528 school-going adolescents for analysis. Adolescent students included in the final sample showed a slightly lower prevalence of overweight and obesity, as well as daily soft drink consumption, compared with those who were excluded. Only minor differences were observed in terms of age, percentage of female students, consumption of vegetables and fruits, or prevalence of physical activity, despite all *P* values being less than .001 (eTable 2 in Supplement 1).

### Soft Drink Consumption

In the previously mentioned data sources, soft drink refers to carbonated beverages that typically contain sugar, including but not limited to brands such as Coca-Cola and Pepsi, as well as nonbrand carbonated beverages with sugar, depending on the country-specific context (eTable 1 in Supplement 1). In the present analysis, soft drink consumption was recorded as a dichotomous variable: daily consumption (1 or more times per day) and nondaily consumption (never or less than 1 time per day)

### Overweight and Obesity

In the GSHS, trained survey staff conducted direct measurements of body weight and height for each student, while the HBSC (Europe) study and the YRBS (US) relied on self-reported data for body weight and height. Body mass index (BMI) was calculated as weight in kilograms divided by height in meters squared. Students were classified as overweight if their BMI values were more than 1 SD above the age- and sex-specific median, and as obese if they were more than 2 SDs above the median.^14^

### Covariates

Based on the data available in the 3 previously mentioned surveys and a literature review of risk factors of obesity,^15^ the covariates included age, sex, daily fruit consumption, daily vegetable consumption, physical activity, soft drink taxes, country income groups defined by the World Bank, and year of data collection. Daily fruit consumption and daily vegetable consumption were defined as eating fruit or vegetables 1 or more times per day. Physical activity was defined as students who were physically active for a total of at least 60 minutes per day on 5 or more days during the past 7 days. The countries that implemented soft drink taxes were identified based on a previous publication,^16^ and the World Bank website was consulted to determine the country income groups for each included countries.^17^

### Statistical Analysis

For the country-level data analysis, the prevalence of adolescent students consuming soft drinks 1 or more times per day, prevalence of overweight and obesity among students, mean (SD) of age, percentage of female students, prevalence of students eating fruit 1 or more times per day, prevalence of students eating vegetables 1 or more times per day, and prevalence of physical activity among students, were estimated using data from surveys of school-going adolescents. To ensure that the data analysis accurately represented the school-going adolescent population in each country, we used sample weights and accounted for the complex study design measures of stratification and clustering. We first examined the partial correlation coefficient between the prevalence of daily soft drink consumption and the prevalence of overweight and obesity, adjusting for covariates, including the mean (SD) age, percentage of female students, prevalence of daily fruit consumption, prevalence of daily vegetable consumption, prevalence of physical activity, soft drink taxes (implemented or not implemented), country income groups (low-, middle-, or high-income countries), and year of data collection. Each country had the same weight. Then, we used linear regression models to investigate the association between the prevalence of daily soft drink consumption and the prevalence of overweight and obesity across countries. In model 1, we adjusted for the mean age and percentage of female students in each country. In model 2, we additionally adjusted for the prevalence of daily fruit consumption, prevalence of daily vegetable consumption, prevalence of physical activity, soft drink taxes, country income groups, and year of data collection. A sensitivity analysis was performed after excluding 2 countries with the highest prevalence of daily soft drink consumption among school-going adolescents, as well as 3 countries with the lowest prevalence of daily soft drink consumption.

In the pooled analysis using individual-level data, the logistic regression model (SAS/STAT SURVEYLOGISTIC Procedure) was used to analyze the association between daily soft drink consumption and overweight and obesity, taking into account survey design elements, such as weights, clusters, and strata. Population-scaled weight was used to account for differences in adolescent population sizes and allow for pooling of data.^18^ Adolescent population size was obtained from the Global Burden of Disease Study 2019 Population Estimates 1950 to 2019.^19^ Country was defined as a cluster variable. We adjusted for individual-level factors, such as age (years), sex (male or female), daily fruit consumption (yes or no), daily vegetable consumption (yes or no), and participation in physical activity (yes or no), as well as country-level factors, such as country income groups, implementation of soft drink taxes, and year of data collection. All of the statistical analyses were performed using SAS version 9.3 (SAS Institute). Statistical significance was established as 2-sided *P* < .05.

## Results

Among the 107 countries and regions included in our analysis, 65 were low- and middle-income, and 42 were high-income countries and regions, with a total of 405 528 school-going adolescents (mean [SD] age, 14.2 (1.7) years; 48.4% males). The country-level characteristics were provided in eTable 3 in Supplement 1. Thirty-two countries (14 low- and middle-income and 18 high-income countries) have implemented taxes on sugar-sweetened soft drinks. High-income countries were more likely to implement soft drink taxes compared with low- and middle-income countries (18 of 42 [42.9%] vs 14 of 65 [21.5%]; *P* = .02). Approximately 17.2% (95% CI, 17.0%-17.5%) of all the school-going adolescents were overweight or obese, and the population-weighted prevalence of overweight and obesity among adolescent students in countries and regions with soft drink taxes was marginally higher compared with those in countries and regions without soft drink taxes (17.4% [95% CI, 17.1%-17.7%] vs 16.3% [95% CI, 15.9%-16.8%]; *P* = .05). The population-weighted prevalence of adolescent students who consumed soft drinks 1 or more times per day was 32.9% (95% CI, 32.3%-33.4%), and the weighted prevalence of daily soft drink consumption was lower in countries with soft drink taxes compared with those without (30.2% [95% CI, 29.6%-30.8%] vs 33.5% [95% CI, 32.8%-34.1%]; *P* <.001).

### Country-Level Data Analysis

As shown in eTable 3 in Supplement 1, the prevalence of overweight and obesity among school-going adolescents varied from 3.3% (95% CI, 2.6%-4.1%) in Cambodia to 64.0% (95% CI, 57.0%-71.6%) in Niue. The prevalence of school-going adolescents consuming soft drinks one or more times per day varied from 3.3% (95% CI, 2.9%-3.7%) in Iceland to 79.6% (95% CI, 74.0%-85.3%) in Niue. There was a positive correlation between the prevalence of daily soft drink consumption and the prevalence of overweight and obesity, with a partial correlation coefficient of 0.44 (95% CI, 0.26-0.58; *P* < .001) (Figure). The country-level prevalence of overweight and obesity among school-going adolescents increased by 3.7% (95% CI, 2.2%-5.2%) for an increase of 10% in the prevalence of daily soft drink consumption (eTable 4 in Supplement 1), after adjusting for multiple covariates. The prevalence of daily soft drink consumption accounted for 12.4% (95% CI, 3.0%-25.8%) of the variation of the prevalence of overweight and obesity among countries. The results did not change materially after excluding 2 countries with the highest prevalence of daily soft drink consumption among school-going adolescents and 3 countries with the lowest prevalence (eFigure 2 and eTable 5 in Supplement 1).

**Figure.**
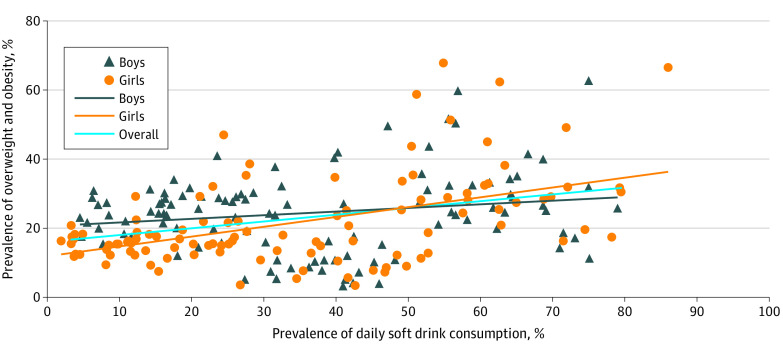
Prevalence of Overweight and Obesity by Soft Drink Consumption Across 107 Countries and Regions in the 2009-2017 Global School-Based Student Health Survey, 2017-2018 European Health Behavior in School-Aged Children, and 2019 US National Youth Risk Behavior Surveys Data are from the Global School-Based Student Health Survey (2009-2017), the European Health Behavior in School-Aged Children study (2017-2018), and the US Youth Risk Behavior Survey (2019). Consumption is the prevalence of daily soft drink consumption (1 or more times per day) among school-going adolescents. The partial correlation coefficient (each country has the same weight) is 0.44 (*P* < .001) for all students, 0.36 (*P* < .001) for males, and 0.50 (*P* < .001) for females, controlling for the mean age, percentage of female students (for the overall analysis), prevalence of daily fruit consumption, prevalence of daily vegetable consumption, percentage of physical activity, implementation of soft drink taxes, country income groups, and year of data collection.

### Individual-Level Data Analysis

The analysis of individual-level data showed that school-going adolescents who consumed soft drinks 1 or more times per day had a higher prevalence of overweight and obesity, with an odds ratio (OR) of 1.14 (95% CI, 1.08-1.21) (Table), compared with those who did not consume soft drinks daily. We observed a slightly weaker association between daily soft drink consumption and overweight and obesity among school-going adolescents in countries with soft drink taxes (OR, 1.09; 95% CI, 1.01- 1.18) compared with those without (OR, 1.15; 95% CI, 1.08- 1.23), but the interaction term was not statistically significant (*P* for interaction = .28).

**Table.** Individual-Level Analysis of the Association Between Daily Soft Drink Consumption and Overweight and Obesity Among School-Going Adolescents^a,b^

Variable	No. of obese and overweight cases/total No. of students (%)	Odds ratio (95% CI)	*P* value for interaction
Overall	86 338/405 528 (21.3%)	1.14 (1.08-1.21)	0.28
With soft drink taxes	31 461/131 439 (23.9%)	1.09 (1.01-1.18)	
Without soft drink taxes	54 877/274 089 (20.0%)	1.15 (1.08-1.23)	

^a^ Adjusted for age, sex, daily fruit consumption, daily vegetable consumption, physical activity, implementation of soft drink taxes (for the overall analysis), country income groups, and year of data collection.

^b^ Data are from the Global School-Based Student Health Survey (2009-2017), the European Health Behavior in School-Aged Children study (2017-2018), and the US Youth Risk Behavior Survey (2019).

## Discussion

To the best of our knowledge, our study is the first to examine the association between soft drink consumption and overweight and obesity among school-going adolescents at both country and individual levels. Using nationally representative data of adolescent students from 107 countries and regions, our study found a statistically significant positive association. For every 10% increase in the prevalence of daily soft drink consumption, there was a 3.7% increase in the prevalence of overweight and obesity after adjusting for potential confounding factors. Furthermore, using individual-level data, our study also found a statistically significant association between daily consumption of soft drinks and overweight and obesity among school-going adolescents.

We observed a large variation in the prevalence of overweight and obesity among school-going adolescents across countries, which was consistent with other studies.^20,21,22^ Our findings of the positive association of daily soft drink consumption with prevalence of overweight and obesity were also supported by prospective cohort studies and randomized clinical trials in children and adolescents.^7,8,9^ For instance, a cohort study conducted by Ludwig et al^23^ among 548 ethnically diverse schoolchildren (mean [SD] age, 11.7 [0.8] years) found that both baseline consumption of sugar-sweetened beverages and a change in consumption were independently associated with BMI change during follow-up. For baseline consumption of sugar-sweetened drinks, BMI increased by 0.18 for each serving consumed per day. For the change in consumption of sugar-sweetened beverages, BMI increased by 0.24 for each additional serving of sugar-sweetened drinks consumed. James et al^24^ carried out a 1-year cluster randomized clinical trial in 6 primary schools with 644 students aged 7 to 11 years. The intervention was to reduce the consumption of carbonated drinks. It was found that the percentage of children with overweight and obesity increased by 7.5% in the control group at 12 months, compared with a decrease of 0.2% in the intervention group. These different types of studies^23,24^ strongly support that reducing soft drink consumption could play an important role in preventing adolescent overweight and obesity worldwide.

Some potential mechanisms underlying the association between soft drink consumption and the development of overweight and obesity have been proposed. One possible mechanism is that consumption of soft drinks, which usually contain high levels of added sugar, can lead to excess energy intake and thus promotes weight gain.^25^ In addition, soft drinks can decrease satiety and result in an incomplete energy intake compensation at subsequent meals following ingestion of liquid calories, which can consequently lead to weight gain.^25,26^

The soft drink tax has been implemented in over 50 countries worldwide to address the growing problems of obesity.^16^ One recent study showed that the UK Soft Drinks Industry Levy, which is a 2-tiered levy, was associated with a decreased prevalence of obesity in year 6 female students.^27^ In the present study, we found that high-income countries were more likely to implement soft drink taxes compared with low- and middle-income countries (42.9% vs 21.5%). Furthermore, in countries with soft drink taxes, the prevalence of daily soft drink consumption among school-going adolescents was lower than in countries without such taxes (30.2% vs 33.5%). These findings suggest that governments, particularly those in low- and middle-income countries, should take actions such as levying taxes on soft drinks to lower soft drink consumption or to reduce the amount of sugar consumption from soft drinks, to help curb the rapid increase in obesity. Additional strategies beyond soft drink taxation, such as reducing saturated fat and calorie intake and increasing physical activity, are also necessary to effectively reduce the burden of obesity in the population.

### Strengths and Limitations

The major strengths of our study include the nationally representative data of adolescents enrolled in schools, standardized methods for data collection in each survey, direct comparisons, and dietary intake assessment, such as fruit and vegetable consumption. Our study also has several limitations. First, this is a cross-sectional study. Thus, no causal association can be drawn. Second, the data on food and drink consumption are self-reported, and therefore subject to social desirability bias. People who have overweight and obesity commonly underreport consumption of unhealthy foods and drinks. Therefore, this may lead to an underestimation of the true association, especially in countries with a high prevalence of overweight and obesity. Furthermore, we had no data on the amount of food and drink intake, especially the volumes and types of soft drinks consumed, although diet carbonated soft drinks were excluded during the survey. Third, the HBSC (Europe) study and the YRBS (US) asked about food and drink consumption based on a 7-day recall period, while the GSHS asked for a 30-day recall period. Recall bias may increase with longer periods of recall. Furthermore, the variation in data collection methods for assessing body weight and height among GSHS, HBSC (Europe), and YRBS (US) may introduce measurement bias and impact result accuracy. Fourth, our study results may be affected by residual and unmeasured confounding factors, including eating patterns, family factors, and neighborhoods. Fifth, there was a substantial decrease in sample size for each survey due to missing data, which could introduce bias and affect the generalizability of our findings. Additionally, data were collected from school-attending adolescents. The lack of data from adolescents who did not go to school may further affect the generalizability of our findings.

## Conclusions

Our study found that there was a significant association between the prevalence of daily soft drink consumption and the prevalence of overweight and obesity among school-going adolescents across countries and that the consumption of soft drinks accounted for approximately 12% of the variation in the overweight and obesity rate. In conjunction with the evidence from prospective cohort studies and randomized trials, our findings support that reducing soft drink consumption should be a prioritized approach for curbing the pandemic of overweight and obesity among adolescents.

## Notes

Supplement 1. **eFigure 1.** Flowchart for Inclusion of the GSHS, HBSC (Europe), and YRBS (US) Surveys **eFigure 2.** Prevalence of Overweight and Obesity by Soft Drink Consumption Across 102 Countries and Regions in the 2009-2017 GSHS, 2017/18 HBSC (Europe), and 2019 YRBS (US) Surveys (Sensitivity Analysis) **eTable 1.** Sampling Methods and Data Collection in the 2009-2017 GSHS, 2017/18 HBSC (Europe), and 2019 YRBS (US) Surveys **eTable 2.** Characteristics of Included and Excluded Adolescent Students From the GSHS, HBSC (Europe), and YRBS (US) Surveys **eTable 3.** Country-Level Characteristics in the 2009-2017 GSHS, 2017/18 HBSC (Europe), and 2019, YRBS (US) Surveys **eTable 4.** Country-Level Analysis of the Association Between the Prevalence of Daily Soft Drink Consumption and Prevalence of Overweight and Obesity Across 107 Countries and Regions in the 2009-2017 GSHS, 2017/18 HBSC (Europe), and 2019 YRBS (US) Surveys Using Multivariate Linear Regression Models **eTable 5.** Country-Level Analysis of the Association Between the Prevalence of Daily Soft Drink Consumption and Prevalence of Overweight and Obesity Across 102 Countries and Regions in the 2009-2017 GSHS, 2017/18 HBSC (Europe), and 2019 YRBS (US) Surveys Using Multivariate Linear Regression Models (Sensitivity Analysis)

Supplement 2.
Data Sharing Statement
