# Supplementary material for: Consumption of Soft Drinks and Overweight and Obesity Among Adolescents in 107 Countries and Regions
Source: JAMA Netw Open. 2023 Jul 24;6(7):e2325158. doi: 10.1001/jamanetworkopen.2023.25158 (PMC10366702; doi:10.1001/jamanetworkopen.2023.25158)
Supplement: Supplement 1. — eFigure 1. Flowchart for Inclusion of the GSHS, HBSC (Europe), and YRBS (US) Surveys eFigure 2. Prevalence of Overweight and Obesity by Soft Drink Consumption Across 102 Countries and Regions in the 2009-2017 GSHS, 2017/18 HBSC (Europe), and 2019 YRBS (US) Surveys (Sensitivity Analysis) eTable 1. Sampling Methods and Data Collection in the 2009-2017 GSHS, 2017/18 HBSC (Europe), and 2019 YRBS (US) Surveys eTable 2. Characteristics of Included and Excluded Adolescent Students From the GSHS, HBSC (Europe), and YRBS (US) Surveys eTable 3. Country-Level Characteristics in the 2009-2017 GSHS, 2017/18 HBSC (Europe), and 2019, YRBS (US) Surveys eTable 4. Country-Level Analysis of the Association Between the Prevalence of Daily Soft Drink Consumption and Prevalence of Overweight and Obesity Across 107 Countries and Regions in the 2009-2017 GSHS, 2017/18 HBSC (Europe), and 2019 YRBS (US) Surveys Using Multivariate Linear Regression Models eTable 5. Country-Level Analysis of the Association Between the Prevalence of Daily Soft Drink Consumption and Prevalence of Overweight and Obesity Across 102 Countries and Regions in the 2009-2017 GSHS, 2017/18 HBSC (Europe), and 2019 YRBS (US) Surveys Using Multivariate Linear Regression Models (Sensitivity Analysis) [file jamanetwopen-e2325158-s001.pdf]

Supplemental Online Content

Hu H, Song J, MacGregor GA, He FJ. Consumption of soft drinks and overweight and obesity among adolescents in 107 countries and regions. *JAMA Netw Open*. 2023;6(7):e2325158. doi:10.1001/jamanetworkopen.2023.25158

eFigure 1. Flowchart for Inclusion of the GSHS, HBSC (Europe), and YRBS (US) Surveys
eFigure 2. Prevalence of Overweight and Obesity by Soft Drink Consumption Across 102 Countries and Regions in the 2009-2017 GSHS, 2017/18 HBSC (Europe), and 2019 YRBS (US) Surveys (Sensitivity Analysis)
eTable 1. Sampling Methods and Data Collection in the 2009-2017 GSHS, 2017/18 HBSC (Europe), and 2019 YRBS (US) Surveys
eTable 2. Characteristics of Included and Excluded Adolescent Students From the GSHS, HBSC (Europe), and YRBS (US) Surveys
eTable 3. Country-Level Characteristics in the 2009-2017 GSHS, 2017/18 HBSC (Europe), and 2019, YRBS (US) Surveys
eTable 4. Country-Level Analysis of the Association Between the Prevalence of Daily Soft Drink Consumption and Prevalence of Overweight and Obesity Across 107 Countries and Regions in the 2009-2017 GSHS, 2017/18 HBSC (Europe), and 2019 YRBS (US) Surveys Using Multivariate Linear Regression Models
eTable 5. Country-Level Analysis of the Association Between the Prevalence of Daily Soft Drink Consumption and Prevalence of Overweight and Obesity Across 102 Countries and Regions in the 2009-2017 GSHS, 2017/18 HBSC (Europe), and 2019 YRBS (US) Surveys Using Multivariate Linear Regression Models (Sensitivity Analysis)

This supplemental material has been provided by the authors to give readers additional information about their work.

© 2023 Hu H et al. *JAMA Network Open.*

eFigure 1 Flowchart for inclusion of the GSHS, HBSC (Europe), and YRBS (US) surveys

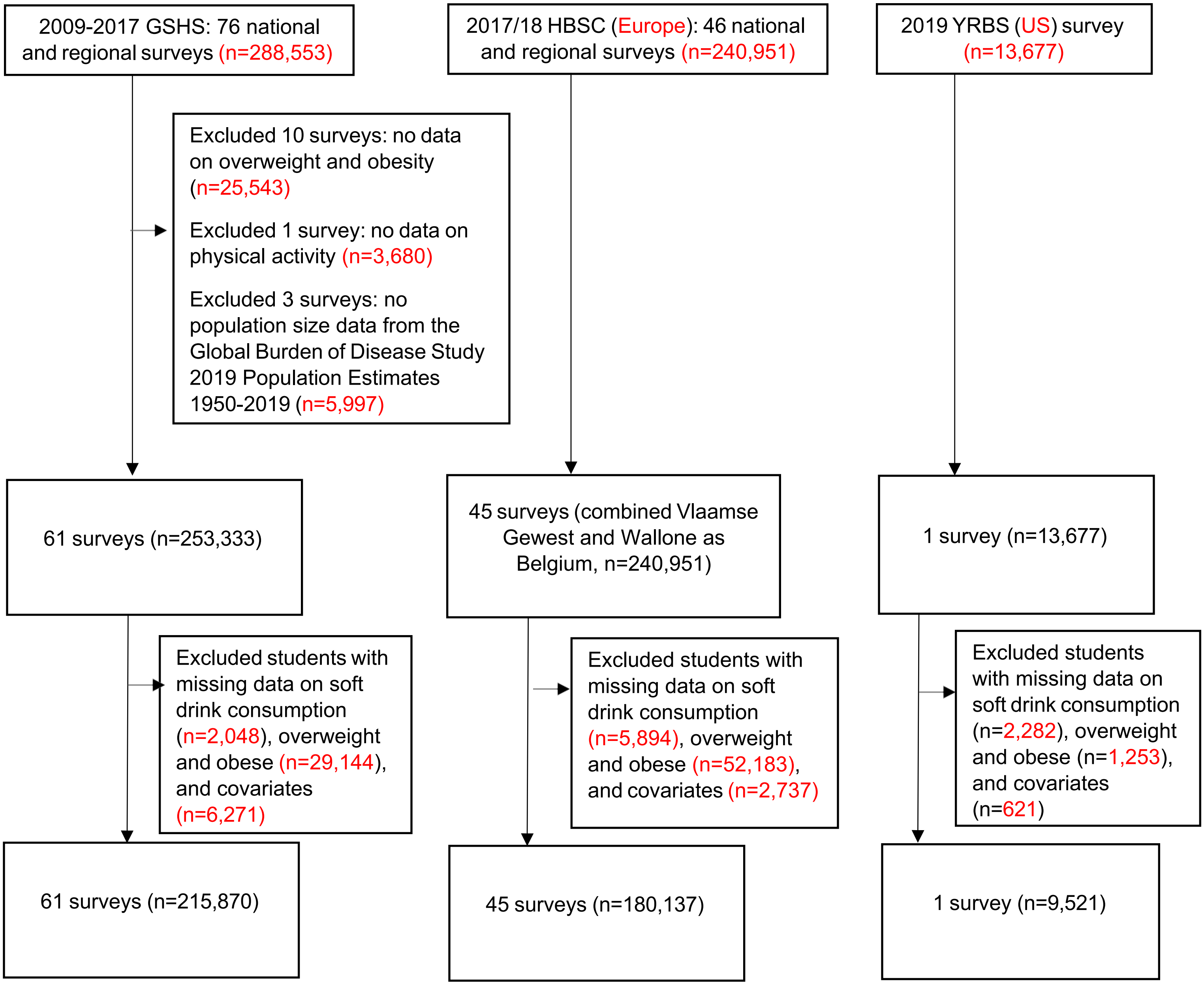

© 2023 Hu H et al. *JAMA Network Open.*

eFigure 2 Prevalence of overweight and obesity by soft drink consumption across 102 countries and regions in the 2009-2017 GSHS, 2017/18 HBSC (Europe), and 2019 YRBS (US) surveys (sensitivity analysis)*

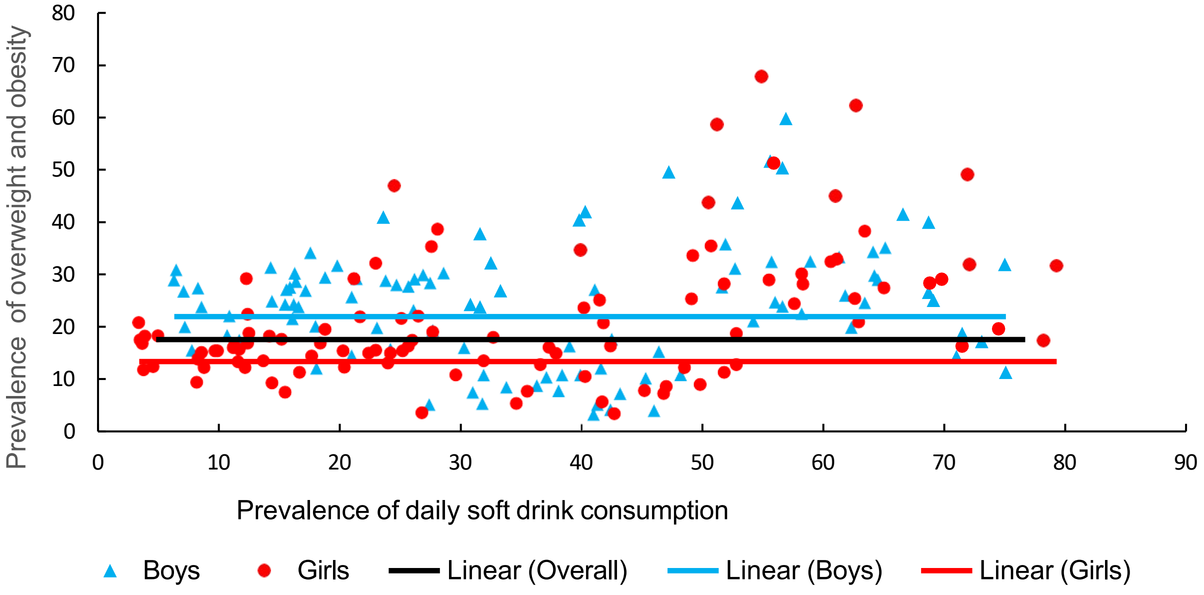

*Two countries with the highest prevalence of daily soft drink consumption among school-going adolescents and three countries with the lowest prevalence of daily soft drink consumption were excluded.

Consumption is the prevalence of daily soft drink consumption (≥ 1 time per day) among school-going adolescents. The partial correlation coefficient (each country has the same weight) is 0.36 (P <0.001) for all students, 0.28 (P =0.006) for male students, and 0.45 (P <0.001) for female students, controlling for the mean age, percentage of female students (for the overall analysis), prevalence of daily fruit consumption, prevalence of daily vegetable consumption, percentage of physical activity, implementation of soft drink taxes, country income groups, and year of data collection.

© 2023 Hu H et al. *JAMA Network Open.*

eTable 1 Sampling methods and data collection in the 2009-2017 GSHS, 2017/18 HBSC (Europe), and 2019 YRBS (US) surveys

	GSHS	HBSC (Europe)	YRBS (US)
**Sampling methods**	A two-stage cluster sampling design is used to obtain a representative sample of students in each country. In the first stage, middle schools were randomly selected by Probability Proportional to Size method. In the second stage, the classes were randomly sampled using systematic sampling method. All students in selected classes were invited to participate in the survey.	The survey employs cluster sampling to select a representative sample of young people aged 11, 13, and 15 in each HBSC country or region, with approximately 1,500 students from each age group. Data are collected through self-administered questionnaires distributed in classrooms.	A three-stage cluster sample design is used to produce a representative sample of students in grades 9-12. In the first stage, primary sampling units (PSUs) such as counties, groups of adjacent counties or parts of larger counties, are categorized into 16 strata based on metropolitan statistical area status and the percentages of non-Hispanic Black and Hispanic students. PSUs are then sampled based on the overall school enrollment size with probability proportional to it. In the second stage, schools are selected with probability proportional to school enrollment. In the third stage, one or two classes from each of grades 9 to 12 are chosen at each school. All students in the selected classes are eligible to participate in the survey. Students complete the self-administered questionnaire during one class period.
**Height and weight**	Measured by trained survey staff	Self-reported	Self-reported

© 2023 Hu H et al. *JAMA Network Open.*

**Soft drink**	During the past 30 days, how many times per day did you usually drink carbonated soft drinks, such as COUNTRY SPECIFIC EXAMPLES? (Do not include diet soft drinks.)<br><br>A I did not drink carbonated soft drinks during the past 30 days<br>B Less than 1 time per day<br>C 1 time per day<br>D 2 times per day<br>E 3 times per day<br>F 4 times per day<br>G 5 or more times per day	How many times a week do you usually drink Coke or other soft drinks that contain sugar?<br><br>1 Never<br>2 Less once a week<br>3 Once a week<br>4 2-4 days a week<br>5 5-6 days a week<br>6 Once daily<br>7 More than once daily	During the past 7 days, how many times did you drink a can, bottle, or glass of soda or pop, such as Coke, Pepsi, or Sprite? (Do not count diet soda or diet pop.)<br>A. I did not drink soda or pop during the past 7 days<br>B. 1 to 3 times during the past 7 days<br>C. 4 to 6 times during the past 7 days<br>D. 1 time per day<br>E. 2 times per day<br>F. 3 times per day<br>G. 4 or more times per day
**Vegetables**	During the past 30 days, how many times per day did you usually eat vegetables, such as<br>COUNTRY SPECIFIC EXAMPLES?<br>I did not eat vegetables during the past 30 days<br>A Less than one time per day<br>B 1 time per day<br>C 2 times per day<br>D 3 times per day<br>E 4 times per day<br>F 5 or more times per day	How many times a week do you usually eat vegetables?<br><br>1 Never<br>2 Less once a week<br>3 Once a week<br>4 2-4 days a week<br>5 5-6 days a week<br>6 Once daily<br>7 More than once daily	During the past 7 days, how many times did you eat green salad, potatoes, carrots, and other vegetables? (Do not count green salad, potatoes, or carrots.)<br>A. I did not eat other vegetables during the past 7 days<br>B. 1 to 3 times during the past 7 days<br>C. 4 to 6 times during the past 7 days<br>D. 1 time per day<br>E. 2 times per day<br>F. 3 times per day<br>G. 4 or more times per day<br><br>Daily vegetable consumption is defined as eating green salad, potatoes, carrots, or other vegetables 1 time or more per day (at least one of the 4 vegetables categories).

© 2023 Hu H et al. *JAMA Network Open.*

**Fruits**	During the past 30 days, how many times per day did you usually eat fruits, such as COUNTRY SPECIFIC EXAMPLES?<br>A I did not eat vegetables during the past 30<br>days<br>B Less than one time per day<br>C 1 time per day<br>D 2 times per day<br>E 3 times per day<br>F 4 times per day<br>G 5 or more times per day	How many times a week do you usually eat fruits?<br><br>1 Never<br>2 Less once a week<br>3 Once a week<br>4 2-4 days a week<br>5 5-6 days a week<br>6 Once daily<br>7 More than once daily	During the past 7 days, how many times did you eat green fruits?<br>A. I did not eat other vegetables during the past 7 days<br>B. 1 to 3 times during the past 7 days<br>C. 4 to 6 times during the past 7 days<br>D. 1 time per day<br>E. 2 times per day<br>F. 3 times per day<br>G. 4 or more times per day<br><br>Daily vegetable consumption is defined as eating green salad, potatoes, carrots, or other vegetables 1 time or more per day (at least one of the 4 vegetables categories).

© 2023 Hu H et al. *JAMA Network Open.*

**Physical activity**	During the past 7 days, on how many days were you physically active for a total of at least 60 minutes per day? ADD UP ALL THE TIME YOU SPENT IN ANY KIND OF PHYSICAL ACTIVITY EACH DAY.<br>A. 0 days<br>B. 1 day<br>C. 2 days<br>D. 3 days<br>E. 4 days<br>F. 5 days<br>G. 6 days<br>H. 7 days	Over the past 7 days, on how many days were you physically active for a total of at least 60 minutes per day? Please add up all the time you spent in physical activity each day.<br><br>0 0 Days<br>1 1 Day<br>2 2 Days<br>3 3 Days<br>4 4 Days<br>5 5 Days<br>6 6 Days<br>7 7 Days	During the past 7 days, on how many days were you physically active for a total of at least 60 minutes per day? (Add up all the time you spent in any kind of physical activity that increased your heart rate and made you breathe hard some of the time.)<br>A. 0 days<br>B. 1 day<br>C. 2 days<br>D. 3 days<br>E. 4 days<br>F. 5 days<br>G. 6 days<br>H. 7 days

© 2023 Hu H et al. *JAMA Network Open.*

eTable 2 Characteristics of included and excluded adolescent students from the GSHS, HBSC (Europe), and YRBS (US) surveys

	Included students	Excluded students	P
N	405,528	137,653	
Mean age (years)	14.2 ±1.7	14.1 ±1.8	<0.001
Prevalence of overweight and obesity	21.4%	27.3%	<0.001
Prevalence of daily soft drink consumption	31.3%	35.9%	<0.001
Percentage of female students	51.6%	51.1%	<0.001
Prevalence of daily fruit consumption	52.8%	51.8%	<0.001
Prevalence of daily vegetable consumption	58.4%	55.3%	<0.001
Prevalence of physical activity	33.2%	32.0%	<0.001
Country income groups			
Low-income countries	4.0%	1.6%	
Lower-middle-income countries	24.6%	12.8%	
Upper-middle-income countries	30.4%	19.4%	
High-income countries	41.0%	66.2%	<0.001
Data source			
GSHS	53.2%	52.8%	
HBSC (Europe)	44.4%	44.2%	
YRBS (US)	2.4%	3.0%	<0.001

© 2023 Hu H et al. *JAMA Network Open.*

eTable 3 Country-level characteristics in the 2009-2017 GSHS, 2017/18 HBSC (Europe), and 2019 YRBS (US) surveys

Country	Prevalence of overweight and obesity	Prevalence of daily soft drink consumption	Mean age (years)	Percentage of female students	Prevalence of daily fruit consumption	Prevalence of daily vegetable consumption	Prevalence of physical activity	Year	Income groups[a]	Soft drink tax	Adolescent population size[b]
Afghanistan	14.7 (11.6,17.9)	37.9 (33.9,42.0)	15.4±0.16	45.9 (30.6,61.2)	57.6 (51.9,63.4)	64.5 (60.1,68.8)	18.2 (15.4,21.1)	2014	low	0	7499077.32
Albania	22.7 (19.2,26.2)	27.1 (24.8,29.4)	13.5±0.10	56.6 (54.0,59.2)	67.5 (65.1,70.0)	47.6 (45.0,50.1)	42.8 (40.6,45.1)	2017	upper middle	0	340105.5479
Algeria	14.5 (12.5,16.4)	76.7 (74.4,79.1)	14.0±0.06	51.8 (50.3,53.3)	82.5 (80.4,84.6)	92.0 (90.5,93.4)	21.1 (19.8,22.3)	2011	upper middle	0	5890621.914
Argentina	27.8 (26.4,29.2)	64 (62.1,65.8)	14.4±0.04	51.3 (49,53.7)	67.0 (65.9,68.0)	73.2 (72.2,74.3)	30.8 (29.5,32.1)	2012	upper middle	0	6441228.06
Armenia	18.4 (17.1,19.7)	27.2 (25.7,28.8)	13.5±0.11	52.5 (50.8,54.3)	63.1 (61.4,64.7)	47.9 (46.3,49.5)	51.9 (50.1,53.8)	2017	upper middle	0	314275.4524
Austria	21.2 (19.9,22.6)	16.7 (15.4,17.9)	13.3±0.09	50.6 (48.3,52.9)	42.3 (40.6,44.1)	32.9 (31.4,34.5)	47.6 (45.7,49.5)	2018	high	0	782930.0113
Azerbaijan	17.2 (15.8,18.6)	13.8 (12.6,14.9)	13.2±0.09	52.7 (51.3,54)	39.3 (37.4,41.2)	34.5 (32.6,36.3)	28.4 (26.8,30)	2017	upper middle	0	1245100.309
Bahamas	45.5 (41.7,49.3)	69.4 (65.2,73.6)	13.5±0.11	53.4 (49.9,57)	54.6 (51.2,57.9)	51.3 (48.1,54.6)	24 (19.5,28.5)	2013	high	0	56627.54331
Bahrain	39.5 (38.3,40.8)	34 (31,36.9)	14.3±0.19	49.9 (37.6,62.3)	51.5 (49.6,53.4)	57.7 (56.1,59.3)	29.2 (26.9,31.6)	2016	high	1	151696.8638
Bangladesh	9.2 (4.8,13.5)	45.8 (42.2,49.5)	14.2±0.09	35.7 (28.3,43.1)	49.6 (45.3,53.9)	74.2 (69.4,79)	54.9 (50.8,58.9)	2014	lower middle	0	28103490.63
Barbados	31.9 (29.1,34.7)	73.6 (71.5,75.7)	14.2±0.09	49.3 (45.4,53.3)	54.3 (51.8,56.9)	61.0 (58.8,63.3)	29.1 (26.8,31.4)	2011	high	1	34560.02804
Belgium	17.1 (16.2,17.9)	25.0 (24.0,26.1)	13.2±0.06	50.9 (49.7,52.2)	45.1 (43.9,46.3)	61.2 (60.1,62.3)	43.3 (42.1,44.5)	2018	high	1	1137685.154
Belize	36.3 (34.1,38.6)	63.7 (60.8,66.7)	13.9±0.13	52.4 (48.8,56)	71.1 (68.5,73.8)	74.5 (72.8,76.2)	31.0 (29.6,32.5)	2011	upper middle	0	69535.33563
Benin	8.3 (6.1,10.4)	34.7 (30.5,38.8)	16.6±0.11	27 (23.7,30.3)	63.5 (58.3,68.8)	64.1 (58.9,69.2)	37.9 (32.4,43.3)	2016	low	0	2441573.315
Bhutan	11.5 (10.4,12.5)	38 (35.6,40.4)	15.6±0.17	51.8 (50.8,52.8)	50.2 (47.0,53.3)	78.8 (77.0,80.5)	23.9 (22.2,25.5)	2016	lower middle	0	129989.5265
Bolivia	22.5 (20.5,24.6)	62.5 (60.6,64.3)	14.4±0.10	48.7 (46.8,50.7)	70.9 (68.8,73)	80.9 (79.1,82.7)	23.8 (21.5,26.2)	2012	lower middle	0	1985993.842

© 2023 Hu H et al. *JAMA Network Open.*

Brunei Darussalam	35.3 (33.2,37.3)	45.8 (43.2,48.5)	14.7±0.10	50.4 (47.0,53.8)	54.2 (52.2,56.1)	65.7 (63.9,67.5)	21.5 (19.9,23.1)	2014	high	0	64870.99908
Bulgaria	23.5 (22.2,24.8)	27.2 (26.0,28.5)	13.6±0.09	52.3 (50.2,54.5)	38.6 (37.3,40.0)	43.3 (41.8,44.7)	48.0 (46.6,49.4)	2018	upper middle	0	586576.5513
Cambodia	3.3 (2.6,4.1)	41.8 (38,45.6)	15.6±0.11	48.6 (46.4,50.7)	52.2 (48.0,56.4)	76.9 (73.9,79.9)	10.6 (9.6,11.7)	2013	low	0	2823266.699
Canada	24.7 (23.5,26)	4.8 (4.2,5.4)	13.9±0.08	51.1 (49.7,52.6)	53.2 (51.6,54.8)	54.3 (52.8,55.7)	62.2 (60.3,64)	2018	high	1	3663762.779
Chile	42.5 (40.2,44.8)	64.8 (60.6,69.1)	15.1±0.14	50.7 (44.8,56.5)	71.4 (68.4,74.4)	84.6 (82.8,86.5)	26.5 (23.9,29.1)	2013	high	0	2273106.67
Cook Islands	63.9 (59.4,68.4)	55.8 (52.6,59.1)	15.4±0.30	51.7 (48.1,55.4)	69 (66.5,71.5)	73.7 (70.8,76.6)	35 (31.3,38.7)	2015	lower middle	1	2610.134088
CostaRica	27.9 (25.6,30.2)	51.7 (48.9,54.5)	14.3±0.08	49.1 (46.8,51.5)	60.3 (58.7,61.9)	74.8 (72.4,77.1)	28.3 (25.8,30.9)	2009	upper middle	0	754934.0825
Croatia	22.4 (21.0,23.7)	16.2 (15.1,17.4)	13.8±0.10	49.1 (47.1,51.2)	34.4 (33.0,35.9)	27.3 (26.0,28.6)	49.8 (48.2,51.4)	2018	high	0	384153.5272
Czech Republic	21.4 (20.5,22.2)	13.6 (12.9,14.4)	13.4±0.07	50.4 (49.5,51.3)	46.1 (45,47.3)	36.9 (35.9,38)	44.3 (43.3,45.4)	2018	high	0	923889.6293
Denmark	13.9 (12.8,15.1)	6.2 (5.4,7.0)	13.4±0.11	50.9 (49.4,52.4)	38.1 (36.4,39.8)	47.2 (45.5,48.9)	33.6 (31.4,35.8)	2018	high	0	610063.4869
Dominica	26.4 (23.8,28.9)	57.1 (54.4,59.7)	14.1±0.17	47.9 (44.0,51.8)	69.1 (66.4,71.8)	69.9 (67.6,72.3)	22.8 (20.3,25.2)	2009	upper middle	1	11527.47586
Dominican Republic	28.4 (25.6,31.2)	74.2 (72.7,75.7)	15.5±0.24	49.8 (46.3,53.4)	59.3 (54.9,63.8)	65 (58.2,71.8)	22.9 (19.4,26.3)	2016	upper middle	0	1751582.15
Egypt	34.0 (31.0,37.0)	54.7 (49.0,60.3)	13.5±0.07	51.3 (41.3,61.4)	79.4 (75.6,83.3)	73.2 (68.4,78)	17.5 (14.1,21)	2011	lower middle	0	15173274.31
El Salvador	28.7 (25.9,31.5)	66.6 (63.4,69.7)	14.3±0.08	48.2 (44.2,52.2)	72.6 (70.5,74.6)	68.5 (65.7,71.4)	21.3 (18.6,23.9)	2013	lower middle	0	1188892.259
England	16.2 (12.6,19.7)	9.5 (5.6,13.3)	15.5±0.03	49.4 (40.4,58.3)	34.9 (29.2,40.5)	41.5 (36,47)	36.7 (31.1,42.4)	2017	high	1	5705723.764
Estonia	22.0 (20.8,23.3)	5.3 (4.6,6)	13.9±0.10	50.9 (49.6,52.2)	41.9 (40.4,43.4)	32.9 (31.5,34.3)	41.9 (40.3,43.5)	2018	high	1	117912.1093
Fiji	29.0 (26.3,31.7)	62.2 (58.1,66.4)	15.7±0.05	52.2 (46.2,58.2)	64 (61.5,66.4)	85.9 (84.4,87.4)	34.5 (31.9,37)	2016	upper middle	1	141988.4379
France	14 (13.2,14.8)	22.3 (21.1,23.5)	13.3±0.08	50.5 (49.3,51.8)	35.8 (34.6,37)	38.6 (37.4,39.7)	29.8 (28.7,31.0)	2018	high	1	7400733.236
Georgia	21.6 (20.0,23.2)	25.8 (24.1,27.6)	13.6±0.12	50.5 (48.6,52.4)	45.2 (43.2,47.2)	37 (35.1,39)	38.1 (35.6,40.6)	2018	lower middle	0	1740694.343

© 2023 Hu H et al. *JAMA Network Open.*

Germany	19.7 (18.4,21.0)	13.7 (12.7,14.8)	13.5±0.10	52.8 (51.1,54.5)	38.3 (36.6,40)	28.2 (26.8,29.7)	36.1 (34.5,37.7)	2018	high	0	7077756.882
Ghana	8.1 (6.2,10.0)	45.4 (42.0,48.7)	15.7±0.14	48.8 (45.5,52.1)	61.6 (58.5,64.7)	72.2 (69.4,74.9)	19.8 (17.4,22.3)	2012	lower middle	0	5431546.281
Greece	24.5 (23.2,25.8)	5.7 (5.0,6.5)	13.9±0.11	51.0 (49.4,52.7)	31.1 (29.5,32.7)	33.7 (32.2,35.1)	42.1 (40.6,43.6)	2018	high	0	933948.4693
Greenland	29.2 (25.4,32.9)	23.8 (20.2,27.3)	13.4±0.13	49.2 (45.3,53.1)	38.3 (34.9,41.8)	37.7 (34.0,41.4)	35.4 (31.5,39.3)	2018	high	0	6680.974542
Guatemala	29.9 (23.9,35.9)	61.4 (57.1,65.8)	14.4±0.19	46.9 (43.6,50.2)	76.7 (72.6,80.8)	76.7 (73.8,79.6)	17.3 (14.8,19.8)	2015	lower middle	0	3378430.715
Guyana	15.3 (13.4,17.3)	71.3 (68,74.5)	14.3±0.05	51.9 (49.4,54.4)	75.2 (72.9,77.5)	83.2 (81.5,84.8)	22.5 (19.0,26.0)	2010	lower middle	0	155905.2076
Honduras	18.5 (15.7,21.3)	73.8 (71.2,76.5)	13.9±0.06	53.3 (50.0,56.6)	67.7 (65.1,70.2)	68.9 (66.6,71.3)	21.6 (19.4,23.8)	2012	lower middle	0	1813011.063
Hungary	25.7 (24,27.3)	24.1 (22.1,26.2)	13.6±0.11	53 (51.1,55.0)	33.1 (31.3,34.8)	28.5 (26.8,30.2)	44.8 (42.8,46.8)	2018	high	1	879915.6569
Iceland	19.7 (18.4,21.0)	3.3 (2.9,3.7)	13.7±0.30	50.2 (49.1,51.4)	41.6 (40.3,42.9)	36.2 (34.6,37.8)	55.8 (54.1,57.6)	2018	high	0	39260.96913
Indonesia	15.7 (14.3,17.1)	27.6 (25.6,29.6)	14.0±0.17	52.1 (50.7,53.5)	63.9 (61.8,66.0)	82.4 (80.9,83.9)	16.3 (15.0,17.5)	2015	lower middle	0	41380394.6
Iraq	24.5 (21.7,27.2)	53.4 (50.8,55.9)	14.4±0.09	42.7 (30.7,54.7)	71.1 (67.8,74.4)	77.4 (74.7,80.1)	20 (17.8,22.2)	2012	upper middle	0	7827364.61
Ireland	15.5 (13.3,17.7)	4.5 (3.3,5.7)	14.0±0.12	41.3 (36.8,45.9)	47.8 (44.7,50.9)	50.4 (47.2,53.5)	58.1 (54.8,61.4)	2018	high	1	591906.6095
Israel	19.8 (18.5,21.2)	25.9 (23.8,28.0)	13.6±0.10	48.6 (46.2,51.1)	47.2 (45.4,48.9)	54.8 (53.3,56.3)	28 (26.4,29.6)	2018	high	0	1336052.481
Italy	23.1 (21.5,24.7)	12.1 (11,13.1)	13.7±0.10	51.4 (49.4,53.3)	36.6 (35,38.3)	27.6 (25.9,29.2)	28.7 (27.1,30.2)	2018	high	0	5168629.916
Jamaica	23.2 (21.3,25.2)	68.1 (63.5,72.6)	15.3±0.14	52.2 (47.2,57.3)	59.5 (56.2,62.9)	66.9 (64.3,69.4)	32.9 (28.6,37.2)	2017	upper middle	0	438711.7439
Kazakhstan	9.8 (8.9,10.7)	16.8 (15.5,18)	13.3±0.10	50.4 (49.1,51.6)	38.2 (36.5,40.0)	43.7 (41.8,45.6)	61.3 (59.7,63.0)	2017	upper middle	0	2247337.927
Kiribati	39.8 (37.1,42.5)	22.3 (19.5,25)	14.3±0.12	52.8 (49.1,56.4)	54 (50.6,57.5)	52.1 (46.6,57.6)	26.5 (23.8,29.2)	2011	lower middle	0	20969.94376
Kuwait	47.7 (44.3,51.2)	53.1 (47.1,59)	15.2±0.13	49.7 (30,69.4)	57.7 (53.7,61.6)	69.3 (66.7,71.9)	25.6 (22.3,28.9)	2015	high	0	401463.2405
Lao Peoples Democratic Republic	11.1 (8.6,13.6)	49.9 (43.4,56.4)	15.8±0.10	46.7 (44.4,49.1)	62.1 (56.7,67.5)	78.2 (75.1,81.2)	22.9 (21.1,24.7)	2015	lower middle	0	1316947.364

© 2023 Hu H et al. *JAMA Network Open.*

Latvia	21 (19.8,22.1)	6.3 (5.6,6.9)	13.5±0.10	50.6 (49.2,51.9)	26.8 (25.4,28.2)	27.2 (25.8,28.5)	41.7 (40.2,43.1)	2018	high	1	168310.4004
Lebanon	25.5 (23.8,27.2)	46.8 (43.8,49.7)	14.8±0.16	54.4 (48.1,60.6)	75.7 (73.8,77.6)	74.4 (72,76.8)	21 (19.2,22.8)	2017	upper middle	0	649558.9505
Lithuania	19.5 (18.2,20.9)	11.9 (10.7,13.1)	13.8±0.19	52.3 (50.5,54)	36.4 (34.6,38.2)	35.3 (33.6,37)	49 (46.7,51.2)	2018	high	0	243625.4801
Luxembourg	23.6 (22.2,25)	22.2 (20.8,23.6)	13.6±0.08	50.3 (48.4,52.1)	38.3 (36.8,39.9)	37.7 (36.1,39.3)	39.7 (38,41.4)	2018	high	0	58844.29994
Macedonia	29.5 (28.2,30.8)	28.2 (26.4,30.1)	13.6±0.10	51.3 (49.5,53.1)	44.7 (43.2,46.3)	46.1 (44.6,47.6)	56 (54.1,57.9)	2018	upper middle	0	214155.9062
Malaysia	22.9 (22.2,23.6)	29 (27.8,30.3)	15.0±0.02	50.3 (48.7,51.9)	67.8 (66.7,69.0)	80.7 (79.8,81.5)	22.7 (21.7,23.8)	2012	upper middle	1	4972035.566
Malta	36.5 (34.2,38.9)	23.3 (20.8,25.8)	13.5±0.21	50.4 (44.4,56.4)	39.1 (36,42.2)	26.4 (23.8,28.9)	41.7 (38.4,44.9)	2018	high	0	36588.72222
Mauritania	21.6 (16.7,26.6)	50.6 (46.1,55.1)	14.8±0.10	46.5 (40.5,52.4)	57.4 (53.3,61.5)	65.5 (60.5,70.5)	16.8 (12.6,21.1)	2010	lower middle	0	688582.4354
Mauritius	25.2 (23.1,27.3)	40.6 (38.0,43.2)	14.9±0.08	53.8 (39.5,68.1)	60.1 (57.5,62.7)	87 (85.2,88.9)	29.3 (26,32.7)	2017	upper middle	1	163526.6326
Mongolia	10.6 (9.4,11.9)	33.2 (31.4,35.1)	14.7±0.09	51.8 (50.4,53.2)	39.8 (38.0,41.5)	75.9 (73.8,78.0)	36.6 (35.2,38.0)	2013	lower middle	0	425563.9716
Morocco	14.1 (11.7,16.5)	32.2 (29.9,34.6)	14.9±0.25	46.1 (43.9,48.4)	73.2 (71.1,75.4)	83.5 (81.8,85.2)	15.2 (14.2,16.2)	2016	lower middle	1	5660831.394
Myanmar	7.6 (5.2,9.9)	44.3 (41.5,47.2)	14.1±0.11	55.3 (53.7,56.8)	60.3 (57.6,63.0)	87.2 (85.3,89.2)	14.8 (12.7,16.8)	2016	low	0	9061844.176
Namibia	6.5 (4.8,8.1)	46.6 (43.8,49.3)	15.9±0.11	53 (51.3,54.7)	55.9 (53.2,58.7)	58.5 (55.3,61.7)	22.2 (20.4,23.9)	2013	upper middle	0	442561.6754
Nepal	6.4 (4.6,8.3)	32.9 (29.3,36.4)	14.4±0.06	51.3 (49.3,53.3)	47.5 (44.3,50.7)	62.3 (59.2,65.4)	21.2 (17.5,24.8)	2015	low	0	6044816.678
Netherlands	12 (10.9,13.1)	17.8 (16.4,19.2)	13.5±0.10	48.2 (46.4,50.1)	35.3 (33.5,37.1)	47.1 (45.3,49)	51 (49.2,52.8)	2017	high	0	1802415.932
Niue	64.3 (57.0,71.6)	79.6 (74,85.3)	14.2±0.15	42.5 (34.9,50.0)	60 (52.6,67.4)	68.5 (61.5,75.6)	30.7 (23.9,37.5)	2010	upper middle	1	235.8615537
Norway	15.9 (14.3,17.5)	5.5 (4.6,6.3)	13.2±0.12	50.3 (48.6,52.0)	36.9 (34.8,39.0)	39.8 (37.6,42.1)	49.2 (46.8,51.7)	2018	high	1	570800.8914
Pakistan	6.7 (5.6,7.7)	36 (30.5,41.6)	14.2±0.05	38.5 (22.9,54.1)	67.7 (62.2,73.2)	90.2 (88.3,92.0)	15.6 (10.8,20.4)	2009	lower middle	0	36924848.63
Paraguay	25.2 (23.2,27.2)	59.7 (56.7,62.6)	14.9±0.15	51.5 (49.5,53.5)	71.2 (69.2,73.2)	84.8 (83.3,86.2)	27.6 (24.9,30.3)	2017	upper middle	0	1180045.519

© 2023 Hu H et al. *JAMA Network Open.*

Peru	19.9 (17.6,22.2)	53.5 (50.4,56.6)	14.4±0.05	49.1 (44,54.2)	72.3 (68.9,75.7)	75.7 (73,78.4)	24.9 (22.8,27)	2010	upper middle	1	5071238.497
Philippines	9.2 (7.1,11.3)	36.9 (34.5,39.4)	14.6±0.06	51.4 (48.7,54.2)	64 (62.6,65.4)	81.1 (79.7,82.5)	13 (11.1,14.8)	2015	lower middle	1	19272833.13
Poland	21.3 (20,22.5)	16.2 (15.1,17.2)	13.7±0.09	51.3 (49.9,52.7)	38.1 (36.7,39.4)	34.4 (33.2,35.6)	46.6 (44.7,48.4)	2017	high	1	3343911.156
Portugal	24.8 (23.6,26)	14 (13.2,14.9)	13.3±0.08	52.8 (51.5,54.2)	47 (45.6,48.3)	33.9 (32.8,35)	28.8 (27.6,29.9)	2018	high	1	998527.727
Republic of Moldova	13.4 (12.5,14.4)	10 (9.1,10.8)	13.6±0.11	50 (48.7,51.3)	47.9 (46.5,49.4)	42.8 (41.4,44.2)	30.5 (29,32)	2018	lower middle	0	361431.0013
Romania	23.1 (21.7,24.5)	19.9 (18.2,21.6)	13.3±0.11	49.7 (47.7,51.8)	41.2 (39.4,43)	31.8 (30.2,33.5)	33.1 (31.4,34.8)	2018	upper middle	0	1920453.723
Russia	16.9 (15.7,18.0)	9.8 (8.9,10.7)	13.9±0.09	53.1 (51.5,54.6)	35.6 (34,37.2)	34 (32.6,35.4)	32.8 (31.2,34.4)	2018	upper middle	0	13844484.36
Saint Kitts and Nevis	32.9 (30.7,35.0)	60.9 (58.7,63.2)	14.5±0.03	48.7 (46.4,51.0)	58.6 (56.3,60.9)	67.3 (65.1,69.5)	25.2 (23.2,27.2)	2011	upper middle	0	8261.786604
Samoa	51.8 (49.2,54.4)	52 (49,54.9)	14.1±0.06	53.9 (49.0,58.8)	69.9 (68.1,71.7)	69.9 (67.1,72.6)	21.5 (19.0,24)	2011	upper middle	1	39653.01616
Scotland	22.2 (19.7,24.6)	13.2 (11.3,15)	13.9±0.16	48.7 (45.9,51.5)	41.1 (37.2,44.9)	45.2 (41,49.4)	53.2 (50.4,56)	2018	high	1	526854.3192
Serbia	21.9 (20.5,23.3)	22 (20.5,23.5)	14.1±0.12	51.6 (49,54.2)	41.9 (39.9,43.9)	41.3 (39.7,42.9)	57.3 (55.5,59.2)	2018	upper middle	0	940230.5774
Seychelles	27.9 (26.3,29.5)	69.3 (67.1,71.5)	14.0±0.15	51.5 (49.0,53.9)	76.1 (74.1,78.1)	75.5 (73.3,77.6)	25 (23,26.9)	2015	high	1	12587.42131
Slovakia	21.4 (20.2,22.6)	21.3 (20.1,22.4)	13.4±0.08	48.7 (47.2,50.2)	41 (39.4,42.5)	35.8 (34.4,37.2)	51.5 (49.9,53.1)	2018	high	0	483433.8079
Slovenia	22.2 (21.1,23.3)	6.1 (5.4,6.7)	13.6±0.09	48.8 (46.7,50.8)	42.7 (41.2,44.3)	36.9 (35.5,38.3)	53.3 (51.8,54.9)	2018	high	0	170012.3664
Solomon Islands	21.2 (17.5,25)	42 (38.7,45.4)	14.5±0.11	47.1 (42.9,51.3)	67.1 (64,70.2)	81.4 (79.1,83.8)	27.9 (25.2,30.7)	2011	lower middle	0	111532.0456
Spain	20.5 (19.3,21.7)	12.9 (11.8,14)	13.7±0.07	50.9 (49.5,52.3)	37.5 (35.9,39.2)	27.8 (26.3,29.2)	49.4 (47.7,51)	2018	high	1	4206224.979
Sudan	9.6 (7.2,12.1)	38.2 (34.6,41.8)	14.7±0.08	47.5 (31.1,63.9)	53.2 (47.5,58.9)	70.7 (68.2,73.1)	11.8 (9.3,14.3)	2012	lower middle	0	7452691.443
Suriname	28.2 (25.6,30.8)	79.3 (77.6,80.9)	14.8±0.16	51.4 (43.2,59.6)	72.1 (70.5,73.7)	88.1 (86.5,89.7)	24.2 (21.6,26.7)	2016	upper middle	0	88793.74456
Sweden	18.5 (17.1,20.0)	4.5 (3.7,5.2)	13.7±0.12	50.6 (48.9,52.3)	26.2 (24.7,27.7)	44 (42.1,45.8)	43.2 (41.3,45.1)	2017	high	0	1000975.311

© 2023 Hu H et al. *JAMA Network Open.*

Switzerland	15.6 (14.8,16.5)	20.0 (19.0,20.9)	13.5±0.06	49.0 (47.9,50.0)	45.9 (44.7,47)	45.8 (44.6,47.1)	45 (43.7,46.3)	2018	high	0	757926.1783
Syrian Arab Republic	23 (20.8,25.3)	30.6 (27.9,33.3)	13.6±0.09	49 (40.3,57.7)	62.7 (59,66.4)	64.3 (61.7,66.8)	15.1 (13.2,17)	2010	lower middle	0	4279384.31
Thailand	17.2 (15.4,18.9)	55.2 (51.7,58.7)	14.7±0.07	54.7 (52,57.4)	73.4 (71.5,75.4)	85.5 (84.4,86.6)	20.4 (18.5,22.3)	2015	upper middle	1	8128903.031
Timor-Leste	5.5 (3.4,7.5)	41.5 (39.1,43.9)	15.9±0.22	50.1 (48.4,51.7)	45.4 (43.2,47.5)	59.7 (57.5,61.9)	13.2 (11.2,15.1)	2015	lower middle	0	277343.3905
Tonga	56.3 (54.4,58.1)	59.6 (57.9,61.3)	14.2±0.15	49.2 (47.5,50.9)	71.6 (69.9,73.2)	76.1 (74.5,77.6)	32.8 (31.3,34.3)	2017	lower middle	1	20486.12263
Trinidad and Tobago	33.1 (31.3,34.8)	52.2 (48.7,55.8)	14.3±0.06	52.7 (46.3,59.2)	48.4 (45.7,51.1)	67.4 (64.9,69.9)	29.9 (27.7,32.2)	2017	high	0	164426.3583
Turkey	24.4 (23.1,25.7)	14.4 (13.4,15.4)	13.5±0.10	51.2 (49.4,53)	31.7 (30.2,33.1)	23.2 (22.1,24.3)	31.7 (30.1,33.2)	2019	upper middle	0	10926920.59
Tuvalu	50.4 (46.9,54)	51.6 (48.1,55.1)	13.8±0.07	50.7 (47.2,54.3)	63.7 (60.3,67.1)	63.8 (60.4,67.2)	18.7 (16,21.5)	2013	upper middle	1	2028.282948
Ukraine	16.1 (15.3,17)	15.1 (14.2,16.0)	13.5±0.07	49.8 (48.6,51.1)	43.6 (42.3,44.8)	52.5 (51.3,53.7)	46.3 (45,47.5)	2018	lower middle	0	3896328.745
United Arab Emirates	38.5 (35.8,41.2)	33.8 (30.1,37.5)	15.1±0.12	51.3 (42.1,60.6)	56.1 (51.6,60.7)	64.6 (60.7,68.4)	26.8 (24.7,29)	2016	high	1	618051.0185
Vanuatu	14.2 (11.8,16.6)	39.4 (36.9,42.0)	15.1±0.13	50.9 (48.1,53.7)	76 (74,77.9)	77.8 (75.8,79.7)	22 (18.9,25.2)	2016	lower middle	1	53532.54123
Viet Nam	5.8 (4.0,7.7)	30.1 (27.1,33.0)	15.8±0.11	53.4 (51.7,55.1)	71.8 (68.3,75.2)	89.2 (88,90.5)	20.4 (18.7,22.2)	2013	lower middle	0	12923966.94
Wales	21.8 (20.5,23.1)	15.4 (14.0,16.8)	14.3±0.13	41.3 (39.5,43.1)	35.7 (33.8,37.6)	39.3 (37.4,41.3)	48.9 (46.7,51.2)	2017	high	1	316221.1419
Yemen	12.1 (9.2,14.9)	36.1 (30.8,41.4)	15.1±0.17	46.8 (38.9,54.7)	59.1 (53.9,64.3)	71.1 (67.7,74.4)	17.7 (15.1,20.3)	2014	lower middle	0	5803760.443
USA	31.6 (29.1,34.2)	14.9 (13.1,16.8)	16.0±0.03	49.7 (48.3,51.1)	32.7 (30.9,34.6)	29.3 (27.5,31.1)	45.2 (43.3,47.2)	2019	high	0	38628387.73

a, low-income countries, lower-middle-income countries, upper-middle-income countries, and high-income countries.

b, all adolescents in each country.

© 2023 Hu H et al. *JAMA Network Open.*

eTable 4 Country-level analysis of the association between the prevalence of daily soft drink consumption and prevalence of overweight and obesity across 107 countries and regions in the 2009-2017 GSHS, 2017/18 HBSC (Europe), and 2019 YRBS (US) surveys using multivariate linear regression models

	Model 1		Model 2	
	**Coefficient, β**	**P**	**Coefficient, β**	**P**
Prevalence of daily soft drink consumption (%)	0.25	<0.001	0.37	<0.001
Mean age (years)	-3.8	0.01	-0.20	0.90
Percentage of female students (%)	0.28	0.32	0.26	0.33
Prevalence of daily fruit consumption (%)			0.26	0.06
Prevalence of daily vegetable consumption (%)			-0.37	0.001
Prevalence of physical activity (%)			0.11	0.31
Year of data collection			-0.39	0.44
Income groups			2.96	0.06
Soft drink taxes			5.72	0.009
Adjusted R-square	20%		42%	

Model 1 adjusted for the mean age and percentage of female students in each country.

Model 2 adjusted for all variables in model 1 plus the prevalence of daily fruit consumption, prevalence of daily vegetable consumption, prevalence of physical activity, implementation of soft drink taxes (yes/no), income groups (treated as a continuous variable, 1, low-and lower-middle income countries; 2, upper-middle income countries; 3, high income countries), and year of data collection.

© 2023 Hu H et al. *JAMA Network Open.*

eTable 5 Country-level analysis of the association between the prevalence of daily soft drink consumption and prevalence of overweight and obesity across 102 countries and regions in the 2009-2017 GSHS, 2017/18 HBSC (Europe), and 2019 YRBS (US) surveys using multivariate linear regression models (sensitivity analysis)*

	Model 1		Model 2	
	**Coefficient, β**	**P**	**Coefficient, β**	**P**
Prevalence of daily soft drink consumption (%)	0.22	<0.001	0.31	<0.001
Mean age (years)	-3.0	0.05	0.21	0.89
Percentage of female students (%)	0.50	0.08	0.36	0.18
Prevalence of daily fruit consumption (%)			0.35	0.02
Prevalence of daily vegetable consumption (%)			-0.36	0.002
Prevalence of physical activity (%)			0.10	0.35
Year of data collection			-0.39	0.44
Income groups			3.21	0.04
Soft drink taxes			4.61	0.04
Adjusted R-square	17%		40%	

*Two countries with the highest prevalence of daily soft drink consumption among school-going adolescents and three countries with the lowest prevalence of daily soft drink consumption were excluded.

Model 1 adjusted for the mean age and percentage of female students in each country.

Model 2 adjusted for all variables in model 1 plus the prevalence of daily fruit consumption, prevalence of daily vegetable consumption, prevalence of physical activity, implementation of soft drink taxes (yes/no), income groups (treated as a continuous variable, 1, low-and lower-middle income countries; 2, upper-middle income countries; 3, high income countries), and year of data collection.

© 2023 Hu H et al. *JAMA Network Open.*
