# Supplementary material for: Consumption of Soft Drinks and Overweight and Obesity Among Adolescents in 107 Countries and Regions
Source: JAMA Netw Open. 2023 Jul 24;6(7):e2325158. doi: 10.1001/jamanetworkopen.2023.25158 (PMC10366702; doi:10.1001/jamanetworkopen.2023.25158)
Supplement: Supplement 2. — Data Sharing Statement [file jamanetwopen-e2325158-s002.pdf]

Data Sharing Statement

Hu. Consumption of Soft Drinks and Overweight and Obesity Among Adolescents in 107 Countries and Regions. *JAMA Netw Open*. Published July 24, 2023. doi:10.1001/jamanetworkopen.2023.25158

Data
Data available: Yes
Data types: Deidentified participant data
How to access data: The Global School-based Student Health Survey dataset is available at https://www.who.int, the European Health Behavior in School-Aged Children study dataset can be accessed via a request to the Data Management Centre (dmc@hbsc.org), and the US Youth Risk Behavior Survey dataset is available at https://www.cdc.gov/healthyyouth/data/yrbs/data.htm.
When available: With publication

Supporting Documents
Document types: None

Additional Information
Who can access the data: The Global School-based Student Health Survey dataset is available at https://www.who.int, the European Health Behavior in School-Aged Children study dataset can be accessed via a request to the Data Management Centre (dmc@hbsc.org), and the US Youth Risk Behavior Survey dataset is available at https://www.cdc.gov/healthyyouth/data/yrbs/data.htm.
Types of analyses: for any purpose
Mechanisms of data availability: without investigator support
